# Lymphatic Vessel Thrombosis in a Patient with Secondary Lymphedema

**DOI:** 10.1097/GOX.0000000000002268

**Published:** 2019-05-24

**Authors:** Hisako Hara, Makoto Mihara, Rie Ohtomo, Sayuri Tanaka

**Affiliations:** From the * Department of Lymphatic and Reconstructive Surgery, JR Tokyo General Hospital, Tokyo, Japan; † Department of Pathology, JR Tokyo General Hospital, Tokyo, Japan.

## Abstract

Lymphatic thrombosis is rarer than venous thrombosis. This case report describes a patient with secondary lymphedema, who was found to have lymphatic thrombosis during lymphaticovenous anastomosis (LVA). A 51-year-old woman underwent hysterectomy and pelvic lymph node dissection for uterine cancer when she was 48 years old, and lymphedema developed in the left leg soon after the operation. She was diagnosed with lymphedema based on lymphoscintigraphic finding. Preoperative echography showed 2 hypoechoic circles measuring about 0.5 mm in diameter that did not collapse with pressure from the probe, although the veins collapsed with pressure. We diagnosed the 2 hypoechoic circles as lymphatic vessels based on the location and longitudinal continuity. During LVA, we identified 2 parallel white vessels beneath the superficial fascia. After they were cut, white material was extruded. A diagnosis of lymphatic thrombosis was made, and we ligated the lymphatic vessels, closed the wound at this site, and performed LVA at other sites (4 sites in the left and 1 site in the right leg). The postoperative course was uneventful. Histopathological examination showed hyperplasty of fibroblasts and organization in the thrombus. Lymphatic thrombosis is sometimes found in the lymphedema-affected extremities. On retrospective consideration, lymphatic thrombosis can be detected with preoperative echography.

## Abstract 2

Supplemental Digital Content is available in the text.

Lymphatic thrombus is a rarer disease than venous thromboembolism.^1^ There are few reports describing clinical cases of lymphatic thrombosis. We previously reported cases of primary lymphedema caused by lymphatic thrombosis and thrombus formation in acquired lymphangiectasia.^2,3^ Kunze et al.^4^ reported thrombosis in the thoracic duct in cervical lymphedema cases.

Lymphaticovenous anastomosis (LVA) is one of the surgical treatments used for lymphedema. Among various types of surgery, LVA is minimally invasive and can be performed under local anesthesia. The procedure is performed in the superficial subcutaneous tissue.^5,6^ Moreover, LVA does not have a risk of donor site lymphedema, which is sometimes noted in lymph node transfer.^7^ It is reported that the patency rate after LVA is 75% at 12 months postoperatively.^8^

This case report describes a patient with secondary lymphedema, who was found to have lymphatic thrombosis during LVA.

## CASE REPORT

A 51-year-old woman underwent hysterectomy and pelvic lymph node dissection for uterine cancer when she was 48 years old, and lymphedema developed in the left leg soon after the operation. She had one episode of cellulitis. Despite wearing elastic stockings, lymphedema worsened, and she visited our institution at the age of 49 years. She was diagnosed with lymphedema based on lymphoscintigraphic finding. There was a development of collateral lymphatic vessels and dermal backflow in bilateral lower leg (See figure, Supplemental Digital Content 1, which displays lymphoscintigraphic findings. Collateral lymphatic vessels were observed in the bilateral lower legs. Lymphatic function in the left thigh was impaired, http://links.lww.com/PRSGO/B98). She had no allergies or other pertinent medical histories.

**Fig. 1.**
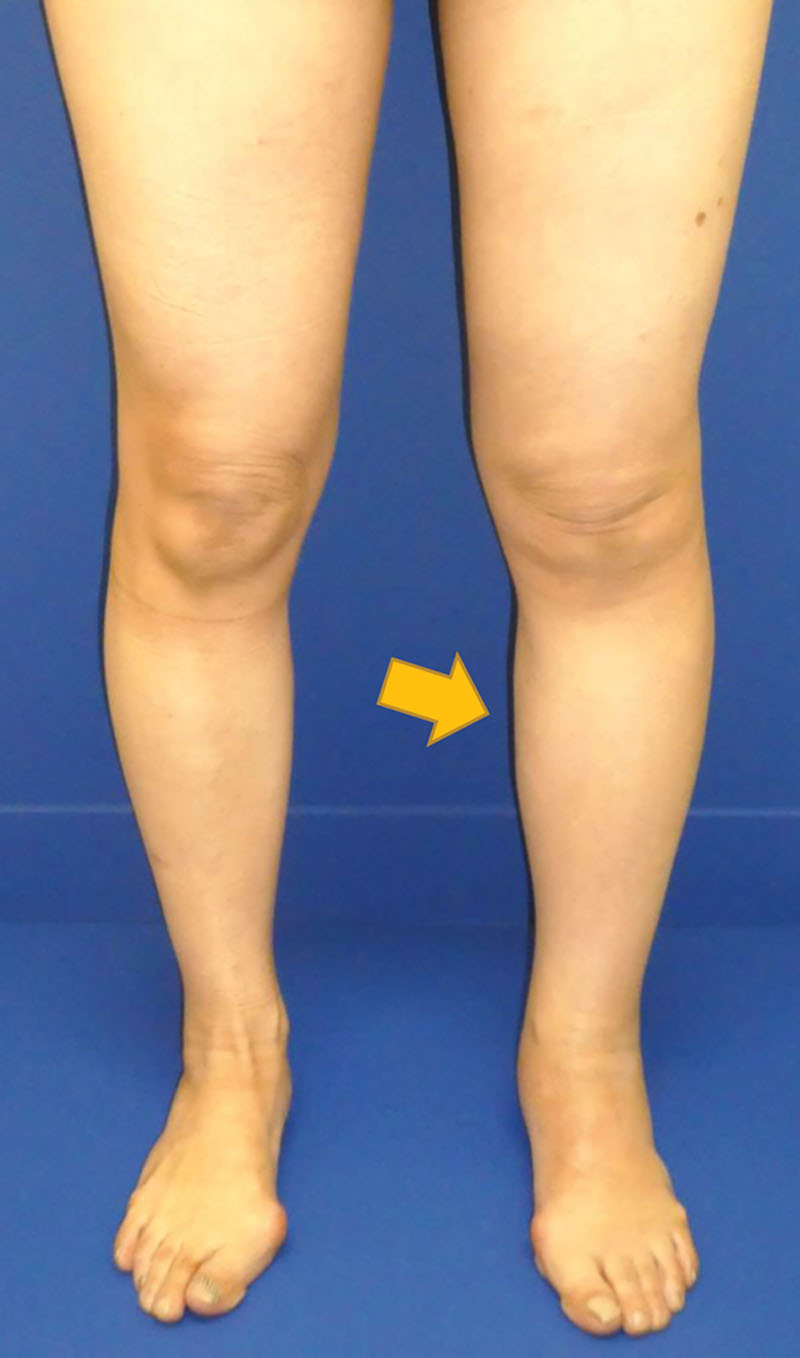
Preoperative clinical appearance. Edema is observed in the left leg. Arrow: site at which lymphatic thrombus was found.

LVA was performed at the age of 50 years. The postoperative course was uneventful, and lymphedema improved. However, lymphedema worsened again at 1 year postoperatively after taking a long flight, although she wears elastic stockings daily. A second LVA was planned (Fig. 1).

**Fig. 2.**
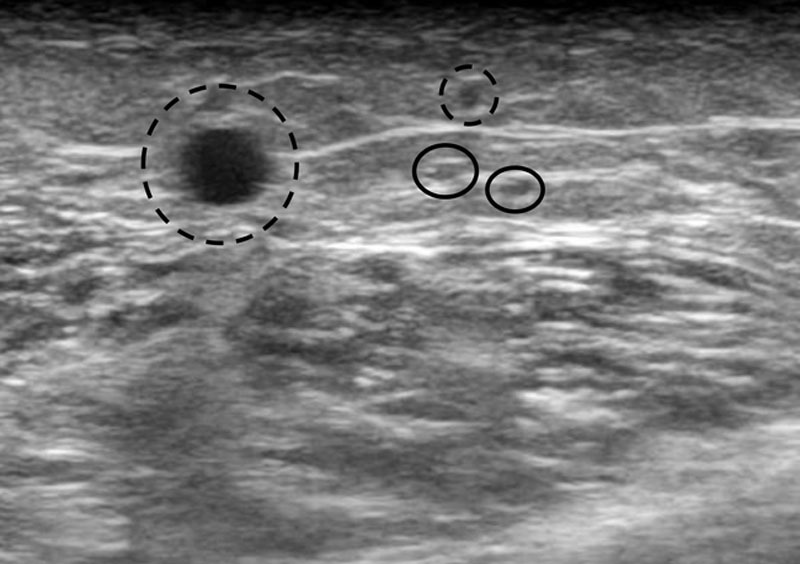
Preoperative echographic finding. Hypoechoic circular objects beneath the superficial fascia indicate lymphatic vessels (circles). Hypoechoic objects above the superficial fascia indicate subcutaneous veins (dotted-lined circles).

Preoperative indocyanine green (ICG) lymphography showed a linear pattern in the right leg. Dermal backflow was observed in the left thigh and lower leg. There was no linear pattern in the area (left lower leg) where the lymphatic thrombus was found afterward. Preoperative echography showed 2 hypoechoic circles measuring about 0.5 mm in diameter that did not collapse with pressure from the probe, although the veins collapsed with pressure (Fig. 2). Compared with lymphatic vessels, veins usually collapse more easily under pressure, because the inner pressure of the lymphatic vessels is higher than that of the veins. In this case, the 2 circles did not collapse under pressure, and we surmised that the inner pressure prevented collapse. The hypoechoic circles extended proximal and distally and did not have flow with on color Doppler mode.

**Fig. 3.**
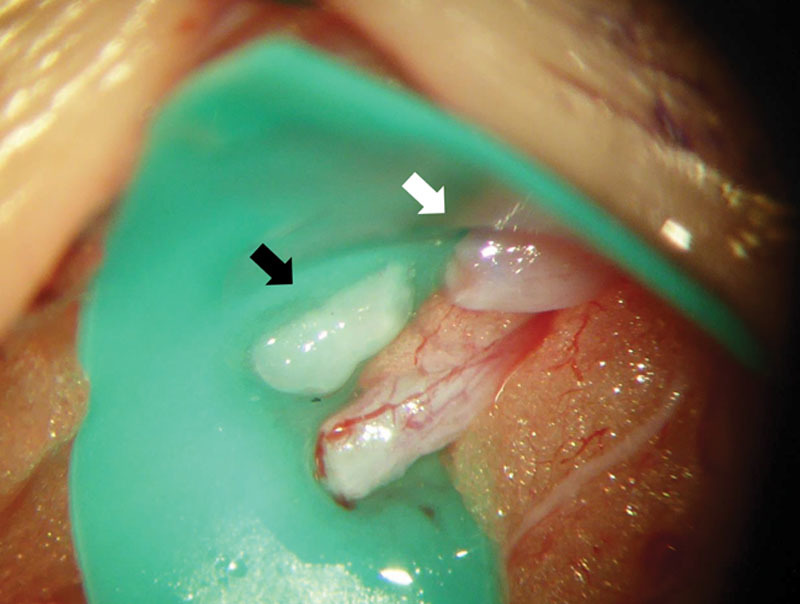
Intraoperative view of lymphaticovenous anastomosis. White material (black arrow) is extruded from the lymphatic vessel (white arrow).

During LVA, we identified 2 parallel white vessels beneath the superficial fascia. Two vessels were in close contact. We diagnosed these as lymphatic vessels because of the location and appearance and the fact that they ran in parallel, which is not usually observed with other vessels or nerves. When we incised the vessels, white material was extruded (Fig. 3). A diagnosis of lymphatic thrombosis was made, and we concluded that the vessels did not collapse with pressure from the probe during echography because of thrombus. Intraoperative echography revealed the same findings, that is, a hypoplastic circle without collapse by probe pressure, 15 cm distal from the incision, which indicated that there was lymphatic thrombus in at least 15 cm. We ligated the lymphatic vessels, closed the wound at this site, and performed LVA at other sites (4 sites in the left and 1 site in the right leg). Though the postoperative course was uneventful, the patient’s lymphedema did not improve postoperatively. This may be partially because the patient gained weight after LVA, and there is another possibility that postoperative thrombus formed within the anastomosis site had harmful effect for lymphedema, although it is difficult to confirm.

**Fig. 4.**
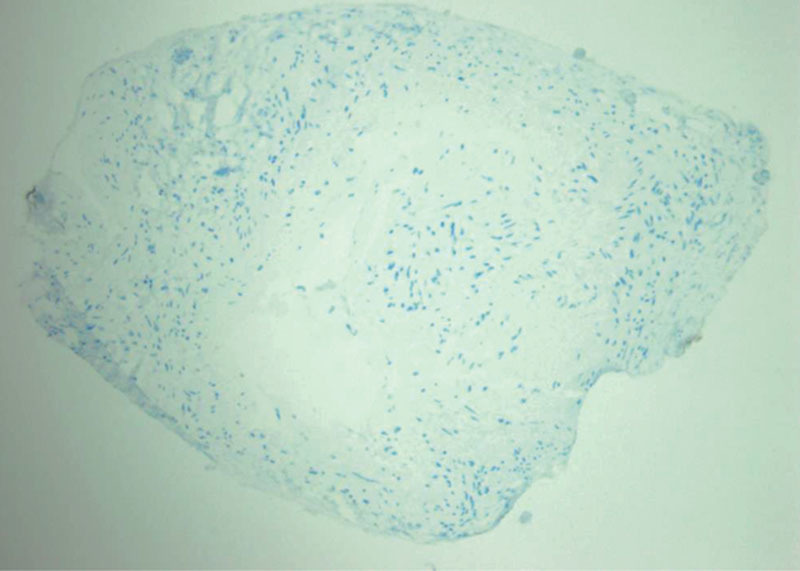
Histopathological findings of the lymphatic thrombus (×100, D2-40). The cells within the thrombus were negative for D2-40.

Histopathological examination showed a thickened smooth muscle layer (tunica media) in the lymphatic vessels (See figure, Supplemental Digital Content 2, which displays histopathological findings of the lymphatic vessel and the lymphatic thrombus. (a) The lymphatic vessel (a) and the lymphatic thrombus (b) (×20, H&E). Thickened smooth muscle layer (tunica media) in the lymphatic vessels is observed. (c) The lymphatic vessel (×100, D2-40). The endothelial cells of the vessel were negative for D2-40, http://links.lww.com/PRSGO/B99). Fibrous thickening of the tunica intima was observed, and the inner lumen was narrow. The inner layer of the vessel was negative for D2-40, which is a marker to stain the lymphatic endothelial cells. In the thrombus, hyperplasty of fibroblasts and organization were found (Fig. 4). We did not observe hyperplasty of the lymphatic endothelial cells, which are positive for D2-40 within the thrombus.

## DISCUSSIONS

This case report described a patient with secondary lymphedema and intraoperative lymphatic thrombosis. We have only encountered 3 such cases among more than 1000 LVA procedures. There have been no other reports from lymphatic surgeons who have a great deal of experience on LVA.^9,10^

In our previously reported case of primary lymphedema, the thrombus consisted of lymphatic endothelial cells. Sclerosis accompanied by proliferation of secreted smooth muscle cells is observed in the collecting lymphatic vessels in the limbs with lymphedema.^11^ A mechanism that promotes endothelial cell proliferation may be present in lymphatic vessels in patients with lymphedema. In contrast, in a case of acquired lymphangiectasia, we found a lymphatic thrombus consisting of fibrin.^3^ Although the clotting function of lymph is said to be less than that of blood,^11^ clot may develop in association with slow flow in cases of acquired lymphangiectasia or occluded lymphatic vessels. In the present case, we did not observe hyperplasty of the lymphatic endothelial cells, and proliferation of the fibroblast was found, which is similar to the general blood thrombus. The causes of lymphatic thrombosis in the current case and the previously reported case may have been different, considering the differences in the histopathological findings.

There have been some studies on the usefulness of echography in observing the lymphatic vessel.^12–14^ The characteristic appearance of a lymphatic vessel on echography is that of a hypoechoic circular object that continues longitudinally.^15,16^ The vessels are located beneath the superficial fascia and do not color under Doppler mode. They are not connected to the subcutaneous veins when traced proximally or distally. As the inner pressure of the lymphatic vessels is higher than that of the veins in lymphedema-affected extremities, they are more difficult to compress with pressure from the probe. Moreover, the echographic findings of lymphatic vessels vary according to the degree of lymphatic sclerosis.^16^ Although the current case was unusual in that the lymphatic vessels found beneath the superficial fascia did not collapse under probe pressure, we diagnosed them as lymphatic vessels because they satisfied the other criteria.

On the contrary, we have to consider the possibility that what we found intraoperatively in the current case was venous thrombus. However, it was located beneath the superficial fascia and was not connected to the thicker veins nearby in echography. Besides, 2 vessels were in close contact with the wound, which is sometimes noted in the lymphatic vessels but unusual in the veins.

In the current case, the lymphatic endothelial cells were not positive for D2-40. However, as previously reported, the lymphatic endothelial cells are negative for D2-40 when the lymphatic vessels degenerate with progression of lymphedema. Therefore, the fact that the endothelial cells of the vessel are negative for D2-40 does not necessarily mean that the vessel is not a lymphatic vessel.^15^

In ICG lymphography, we did not find a linear pattern in the area where a lymphatic thrombus was found afterward. A situation wherein there is no linear pattern in ICG lymphography but a hypoechoic circle is observed in echography sometimes occurs in the clinical setting. Usually, a dilated lymphatic vessel can be found in this situation. In the current case, we came across the lymphatic thrombus, which is an unusual occurrence. It does not mean that all lymphatic surgeons should perform echography preoperatively, but we should be careful when the hypoechoic circle does not collapse with the pressure of the probe, because it may indicate the presence of lymphatic thrombus.

Based on our experience, lymphatic thrombus can be detected only with echography, not lymphoscintigraphy or ICG lymphography. The findings on lymphoscintigraphy or ICG lymphography are nonspecific and limited to dermal backflow or a lack of linear pattern, which were observed in the current case. Before we began lymphatic echography, we made incisions in areas without linear pattern on lymphoscintigraphy or ICG lymphography, based on the anatomical location of the lymphatic vessels. It was possible that we encountered the lymphatic thrombus in these cases. With lymphatic echography, we can detect the lymphatic thrombus preoperatively and more safely perform LVA.

The causal association between lymphedema and lymphatic thrombosis is unclear. One possibility is that lymphatic thrombosis develops first and induces lymphedema. Another possibility is that lymphatic stasis and lymphedema develop first, with thrombus formation due to low flow in the occluded lymphatic vessels.

Lymphatic thrombosis is sometimes found in lymphedema-affected extremities. Retrospectively, the detection of lymphatic thrombosis was possible with preoperative echography. Careful consideration of noncollapsible hypoechoic circles on preoperative echography is prudent, keeping in mind the entity of lymphatic thrombosis. Future research is necessary on the etiology of lymphatic thrombosis.

## Supplementary Material
