# Supplementary figures and images for: Lymphatic Vessel Thrombosis in a Patient with Secondary Lymphedema
Source: Plast Reconstr Surg Glob Open. 2019 May 24;7(5):e2268. doi: 10.1097/GOX.0000000000002268 (PMC6571332; doi:10.1097/GOX.0000000000002268)

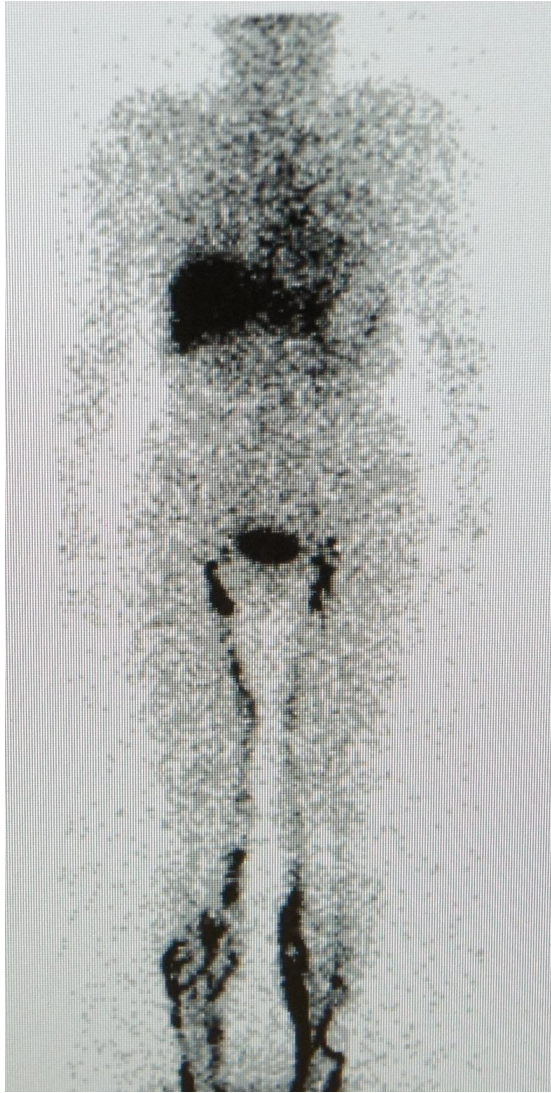

Supplement: Supplementary file 1 [file gox-7-e2268-s001.pdf]

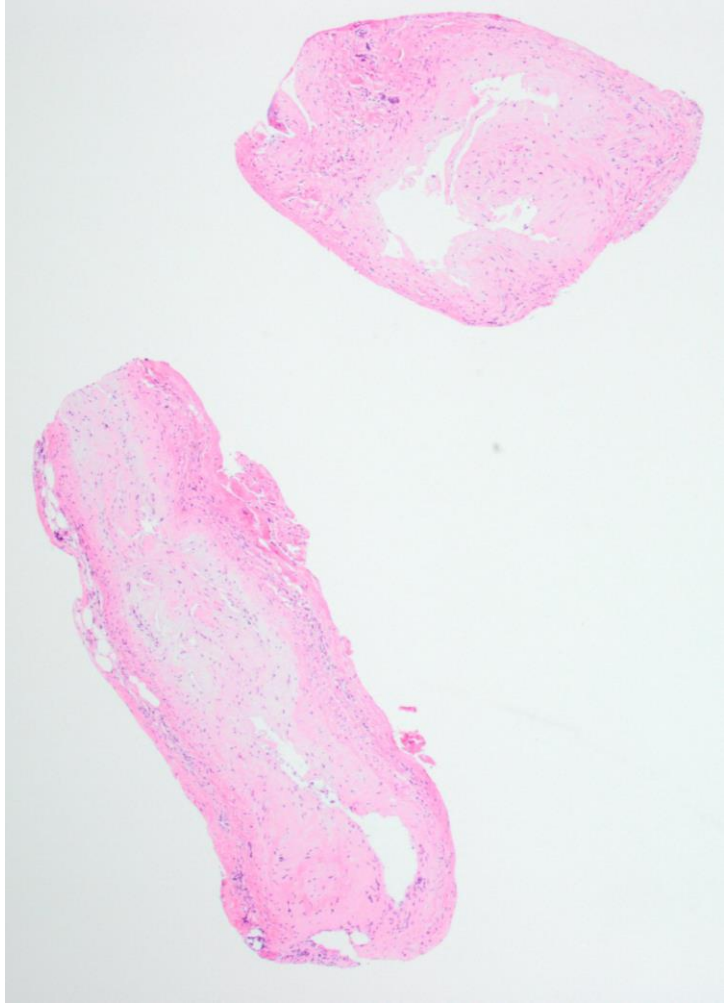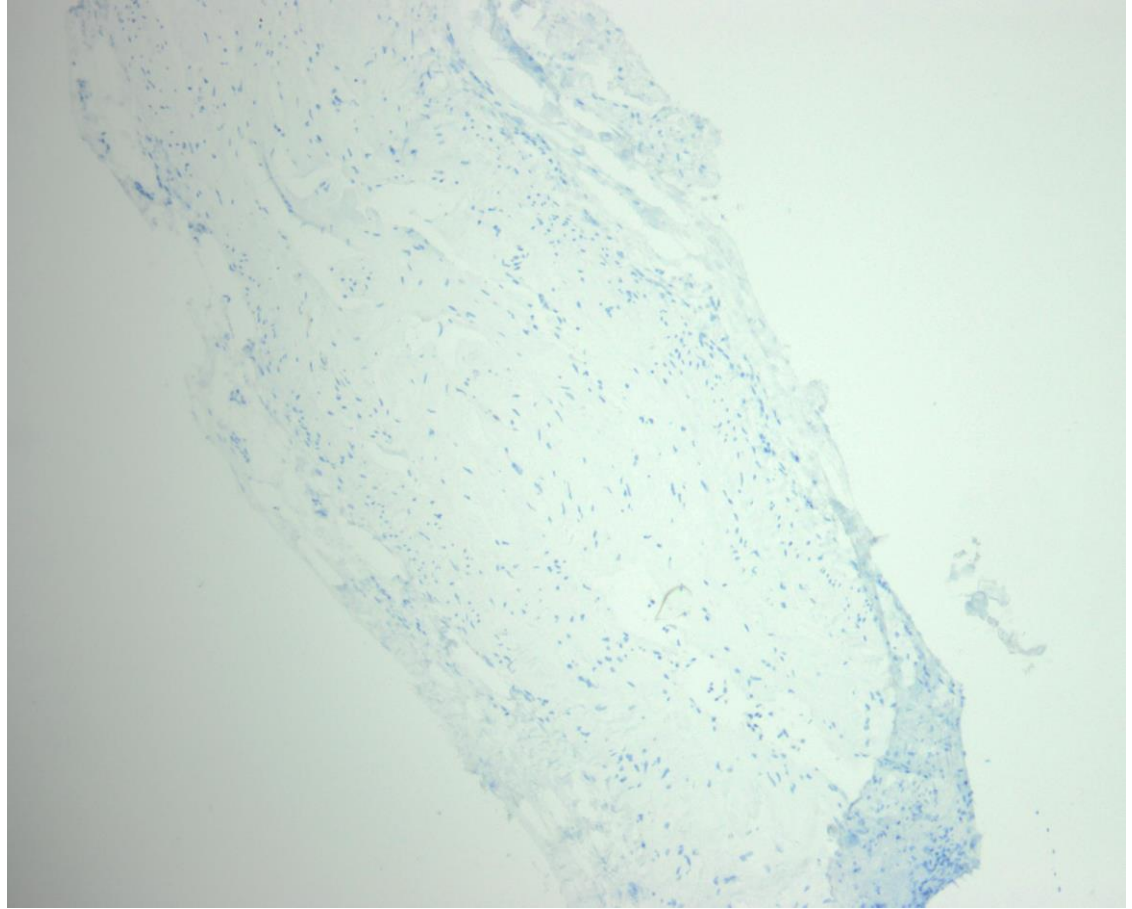

Supplement: Supplementary file 2 [file gox-7-e2268-s002.pdf]
